# Gender difference in heart failure with preserved ejection fraction: clinical profiles, examinations, and prognosis

**DOI:** 10.1007/s00380-022-02067-2

**Published:** 2022-04-24

**Authors:** Takahiro Sakai, Hirohiko Motoki, Sho Suzuki, Aya Fuchida, Takahiro Takeuchi, Kyuhachi Otagiri, Masafumi Kanai, Kazuhiro Kimura, Masatoshi Minamisawa, Koji Yoshie, Tatsuya Saigusa, Soichiro Ebisawa, Ayako Okada, Hiroshi Kitabayashi, Koichiro Kuwahara

**Affiliations:** 1 Department of Cardiovascular Medicine, Ina Central Hospital, Ina, Japan; 2 Department of Cardiovascular Medicine, Shinshu University School of Medicine, 3-1-1 Asahi, Matsumoto, 390-8621 Japan; 3 Department of Cardiovascular Medicine, National Cerebral and Cardiovascular Center, Suita, Japan

**Keywords:** Gender, HFpEF

## Abstract

Heart failure with preserved ejection fraction (HFpEF) has currently become a major concern in the aging society owing to its substantial and growing prevalence. Recent investigations regarding sacubitril/valsartan have suggested that there is a gender difference in the efficacy of the medication in HFpEF cohort. However, information of gender difference in clinical profiles, examination, and prognosis have not been well investigated. The present study aimed to evaluate the differences in baseline characteristics and outcomes between women and men in a Japanese HFpEF cohort. We analyzed the data from our prospective, observational, and multicenter cohort study. Overall, 1036 consecutive patients hospitalized for acute decompensated heart failure were enrolled. We defined patients with an ejection fraction (EF) of ≥ 50% as HFpEF. Patients with severe valvular disease were excluded; the remaining 379 patients (women: *n* = 201, men: *n* = 178) were assessed. Women were older than men [median: 85 (79–89) years vs. 83 (75–87) years, *p* = 0.013]. Diabetes mellitus, hyperuricemia, and coronary artery disease were more prevalent in men than in women (34.8% vs. 23.9%, *p* = 0.019, 23.6% vs. 11.4%, *p* = 0.002, and 23.0% vs. 11.9%, *p* = 0.005, respectively). EF was not significantly different between women and men. The cumulative incidence of cardiovascular death or hospitalization for congestive heart failure (CHF) was significantly lower in women than in men (log-rank *p* = 0.040). Women with HFpEF were older and less often exhibited an ischemic etiology; further, they were associated with a lower risk for cardiovascular death or hospitalization for CHF compared with men in the Japanese population.

## Introduction

Heart failure (HF) is a major concern because of its substantial and growing prevalence and high morbidity in our aging society. A previous study revealed that HF with preserved ejection fraction (HFpEF) has been increasing, and the prevalence of HFpEF is approximately half of the entire HF population [1]. For HFpEF, there is clear evidence of important differences between genders. HFpEF represents the most frequent form of HF in women, with approximately two-fold higher prevalence than in men. In the HF population, women are older than men and have a higher incidence of hypertension and obesity; however, women typically have a lower risk of mortality than men. Several authors have attempted to describe this difference according to comorbidities, which are more prevalent in women [2, 3]; however, the mechanism of gender difference in HFpEF has not completely been evaluated. To date, no medical therapy has been established for HFpEF for improving its mortality or cardiovascular outcomes. However, recent investigations have revealed that the angiotensin receptor–neprilysin inhibitor (ARNI) sacubitril–valsartan reduces the risk of hospitalization for HF or cardiovascular death in patients with reduced ejection fraction (EF) (< 40%) compared with enalapril alone [4]. ARNI did not show a significant association with improving outcomes of HFpEF; however, positive efficacy was indicated in outcomes in women [5]. These results suggested that there were gender differences in terms of the efficacy of medication in HFpEF; it would lead to a better understanding of HFpEF as well as improve gender-specific therapy. The present study aimed to evaluate the differences in baseline characteristics and outcomes between women and men in a Japanese HFpEF cohort.

## Methods

### Patient population

This prospective, observational multicenter cohort study was conducted in Nagano prefecture, Japan. Overall, 1036 consecutive patients admitted for acute decompensated HF (ADHF) were enrolled from July 2014 to December 2018 at 13 institutions in Nagano prefecture, Japan. HF diagnosis was based on the criteria of the Framingham study [6]. Exclusion criteria were as follows: patients aged < 20 years, those for whom follow-up was unfeasible, those from whom obtaining informed consent was difficult, those with acute coronary syndrome (ACS), and those with in-hospital death. The clinical scenario was recorded at admission. Following admission, medical therapy was administered at the discretion of each physician. Patient demographics, past medical history, medications, echocardiogram, electrocardiogram, and laboratory data were obtained at the compensated state of HF before discharge. Echocardiography was conducted in accordance with the recommendations of the American Society of Echocardiography [7]. The left ventricular (LV) EF was measured with the biplane modified Simpson’s method. The e’wave value was obtained from both septal and lateral LV walls, and we calculated the mean value of the E/e’ wave from septal and lateral walls. Following discharge, follow-up data were collected from medical records or telephonic interview. Clinical event was defined as all-cause death, cardiovascular death, acute coronary syndrome, stroke, hospitalization for HF, and hospitalization for any cardiovascular disease.

This study was approved by the Shinshu University Institutional Ethics Committee and was performed in accordance with the 1975 Declaration of Helsinki guidelines for clinical research protocols. Informed consent was obtained from all the patients.

### Study protocol

**Fig.1:**
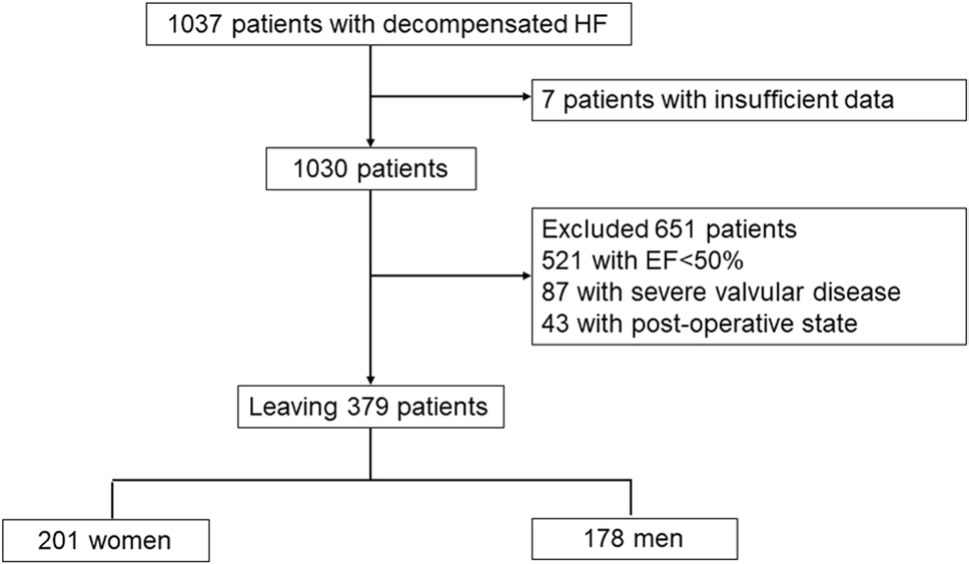
Study flow diagram *EF* ejection fraction, *HF* heart failure

Among 1036 patients, 6 patients with insufficient data, 521 patients with EF of < 50%, 87 patients with severe valvular disease, and 43 patients who were in postoperative state were excluded. The remaining 379 patients (women: 201, men: 178) were diagnosed as HFpEF (Fig. 1). The primary endpoint was composite of cardiovascular death or rehospitalization for HF. The secondary endpoint were cardiovascular death, rehospitalization for HF, and all-cause death.

### Statistical analyses

Continuous variables were expressed as median and interquartile ranges, and categorical variables were expressed as number and percentage. The patients’ baseline characteristics were compared using a contingency table and *t* test for normally distributed continuous variables, the Mann–Whitney *U* test for non-normally distributed continuous variables, and the chi-square test for categorical variables. *P* values of < 0.05 were considered statistically significant. The cumulative incidence of the endpoint was analyzed using the Kaplan–Meier analysis, and differences were assessed with the log-rank test. Cox proportional hazard analysis was performed to adjust for baseline differences and assess whether the variables were independent predictors of outcome. First, covariates that were associated with outcomes in univariate analysis (*p* < 0.05) were included in the multivariate analysis (Model 1). Second, BNP as a conventional risk factor for HF was added to Model 1 instead of loop diuretics use (Model 2). SPSS statistical software, version 27.0 (SPSS Inc., Chicago, Illinois) and R statistical software (The “R” Foundation for Statistical Computing, Vienna, Austria) were used for the analysis.

## Results

### Patient characteristics

Baseline characteristics are presented in Table 1. Women were significantly older than men [85 (79–89) years vs. 83 (75–87) years, *p* = 0.013]. Heart rate was higher in women than in men; however, a higher incidence of diabetes mellitus, hyperuricemia, and coronary artery disease as well as a higher frequency of previous coronary artery bypass graft or percutaneous coronary intervention were noted in men. Higher smoking behavior and incidence of chronic obstructive pulmonary disease (COPD) were observed in men than in women. History of admission for HF was not different between women and men. Living alone was slightly higher in men than women although not statistically different. There were no differences about medication between the two groups. Regarding laboratory data, hemoglobin level was higher in men than in women. However, albumin, brain natriuretic peptide, C-reactive protein, serum potassium, and estimated glomerular filtration rate were not different between women and men. Serum sodium levels were higher in women. In terms of echocardiographic variables, EF and left atrial diameter were not significantly different between women and men; however, left ventricular end-diastolic/systolic diameters were smaller in women than in men. Although the ratio of E wave/A wave and deceleration time were not significantly different, the ratio of E wave/e’ wave value was higher in women than in men.

**Table 1:** Baseline characteristics

Variables	Women (*n* = 201)	Men (*n* = 178)	*p* value
Age (years)	85 (79, 89)	83 (75, 87)	0.013
BMI (kg/m2)	20.8 (18.6, 24.8)	21.0 (19.2, 23.8)	0.951
Heart rate	70 (60, 80)	67 (57, 75)	0.008
Systolic blood pressure	115 (105, 130)	117 (103, 129)	0.831
Diastolic blood pressure	65 (55, 74)	64 (57, 73)	0.849
Clinical scenario			
1, *n* (%)	66 (32.8%)	67 (37.6%)	0.328
2, *n* (%)	117 (58.2%)	96 (53.9%)	0.344
3, *n* (%)	12 (6.0%)	11 (6.2%)	0.932
5, *n* (%)	6 (3.0%)	4 (2.2%)	0.755
Hypertension, *n* (%)	153 (76.1%)	129 (72.4%)	0.417
Dyslipidemia, *n* (%)	54 (26.9%)	43 (24.2%)	0.547
Diabetes mellitus, *n* (%)	48 (23.9%)	62 (34.8%)	0.019
Hyperuricemia, *n* (%)	23 (11.4%)	42 (23.6%)	0.002
Atrial fibrillation, *n* (%)	111 (55.2%)	96 (53.9%)	0.760
Coronary artery disease, *n* (%)	24 (11.9%)	41 (23.0%)	0.005
Heart failure, *n* (%)	47 (23.4%)	41 (23.0%)	0.936
Stroke, *n* (%)	28 (13.9%)	28 (15.7%)	0.622
COPD, *n* (%)	3 (1.5%)	24 (13.5%)	< 0.001
Smoking habit, *n* (%)	5 (2.5%)	101 (56.7%)	< 0.001
Malignant tumor, *n* (%)	16 (8.0%)	18 (10.1%)	0.474
Living alone, *n* (%)	21 (10.4%)	27 (15.2%)	0.168
Dementia, *n* (%)	41 (20.4%)	12 (6.7%)	< 0.001
Medication			
Antiplatelet drugs, *n* (%)	58 (28.9%)	65 (36.5%)	0.112
Warfarin, *n* (%)	47 (23.4%)	38 (21.3%)	0.636
DOAC, *n* (%)	72 (35.8%)	63 (35.4%)	0.931
ACEi/ARB, *n* (%)	130 (64.7%)	125 (70.2%)	0.251
Beta blocker, *n* (%)	126 (62.7%)	106 (59.6%)	0.577
MRA, *n* (%)	100 (49.8%)	81 (45.5%)	0.409
Loop diuretics, *n* (%)	168 (83.6%)	142 (79.8%)	0.338
Tolvaptan, *n* (%)	26 (12.9%)	39 (21.9%)	0.021
SGLT2i, *n* (%)	8 (4.0%)	7 (3.9%)	0.981
Statins, *n* (%)	47 (23.4%)	48 (27.0%)	0.422
Laboratory data			
Albumin (g/dL)	3.3 (3.0, 3.7)	3.3 (3.0, 3.7)	0.691
Hemoglobin (g/dL)	11.2 (10.0, 12.6)	11.7 (10.3, 13.5)	0.003
eGFR (ml/min/1.73m^2^)	44.0 (30.3, 55.9)	44.5 (33.6, 57.0)	0.732
BNP (pg/ml)	201 (93, 432)	199 (101, 383)	0.601
CRP (mg/dL)	0.30 (0.10, 0.80)	0.30 (0.12, 0.68)	0.281
Serum sodium (mEq/L)	140 (138, 142)	139 (137, 141)	0.001
Serum potassium (mEq/L)	4.3 (3.9, 4.7)	4.3 (4.0, 4.7)	0.132
Echocardiographic data			
LAD (mm)	4.5 (3.9, 4.9)	4.4 (3.9, 5.0)	0.254
LVEF (%)	63,4 (57.0, 67.9)	60.4 (55.0, 68.0)	0.101
LVDd (mm)	4.4 (4.0, 4.9)	4.7 (4.3, 5.2)	< 0.001
LVDs (mm)	2.8 (2.5, 3.1)	3.0 (2.7, 3.5)	< 0.001
LV mass index (g/m^2^)	123 (100, 149)	124 (102, 155)	0.686
E/A	0.86 (0.66, 1.27)	0.87 (0.64, 1.31)	0.875
Deceleration time (s)	200 (167, 264)	200 (166, 241)	0.243
*E/e*’ mean	13.1 (10.5, 17.5)	12.1 (9.4, 14.8)	0.016
TRPG (mmHg)	27.0 (22.6, 35.9)	28.9 (23.0, 33.7)	0.481

*ACEi* angiotensin-converting enzyme inhibitor, *ARB* angiotensin receptor blocker, *BMI* body mass index, *BNP* brain natriuretic peptide, *COPD* chronic obstructive pulmonary disease, *CRP* C-reactive protein, *DOAC* direct oral anticoagulant, *E/A* ratio of Ewave/Awave, *E/e mean* ratio of Ewave/e’wave, *eGFR* estimated glomerular filtration rate, *LAD* left atrial diameter, *LVEF* left ventricular ejection fraction, *LVDd* left ventricular diameter at end diastole, *LVDs* left ventricular diameter at end-systole, *MRA* mineralocorticoid receptor antagonist, *SGLT2i* sodium glucose cotransporter 2 inhibitor, *TRPG* transtricuspid pressure gradient

### Outcomes

**Fig.2:**
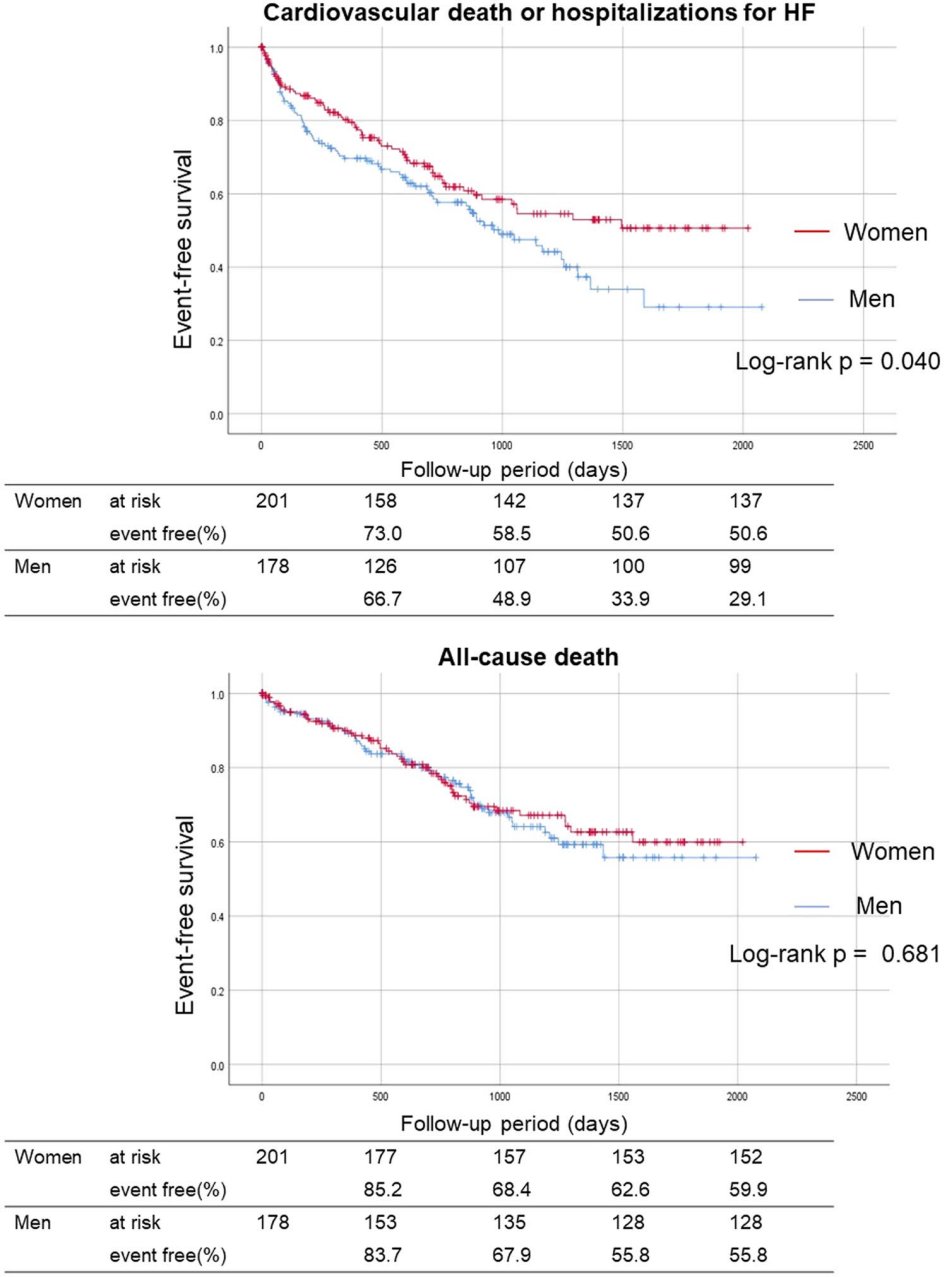
Kaplan–Meier analysis of primary and secondary outcomes compared between women and men. The incidence of cardiovascular death or hospitalization for HF were significantly lower in women than in men. The incidence of all-cause death was not significantly different between women and men

The median follow-up period was 730 (interquartile range: 334–1194) days. There were 54 cardiovascular deaths (women: 27, men: 27), 123 hospitalizations for HF (women: 53, men: 70), and 99 all-cause deaths (women: 49, men: 50) during follow-up period (Table 2). Kaplan–Meier analysis revealed that the cumulative incidence of cardiovascular deaths or hospitalizations for HF were significantly lower in women than in men (log-rank *p* = 0.040). However, the cumulative incidence of all-cause deaths was not significantly different (log-rank *p* = 0.681; Fig. 2). The Cox proportional hazard analysis revealed that female gender was independently associated with a lower risk of cardiovascular death or hospitalizations for HF with HR (Model 1) of 0.64 (95% CI, 0.45–0.91; *p* = 0.012) and HR (Model 2) of 0.64 (95% CI, 0.45–0.93; *p* = 0.018; Table3).

**Table 2:** Incidence of primary and secondary outcomes

	Women 201	Men 178	*p* value
Cardiovascular death or HF hospitalization	64 (31.8%)	79 (44.4%)	0.008
Cardiovascular death	27 (13.4%)	27 (15.2%)	0.691
HF hospitalization	53 (26.4%)	70 (39.3%)	0.005
All-cause death	49 (24.3%)	50 (28.0%)	0.440
Acute coronary syndrome	1 (0.5%)	3 (1.7%)	0.339
Stroke	9 (4.5%)	8 (4.5%)	0.968
Hospitalization for any cardiovascular disease	20 (10.0%)	19 (10.7%)	0.783

*HF* heart failure

**Table 3:** Cox univariate and multivariate analysis for primary outcome

Variables	Unadjusted p value	Unadjusted HR (95% CI)	Model 1		Model 2	
			Adjusted p value	HR (95% CI)	Adjusted p value	HR (95% CI)
Age (years)	< 0.001	1.06 (1.04–1.08)	< 0.001	1.05 (1.02–1.07)	< 0.001	1.05 (1.03–1.08)
Women	0.041	0.71 (0.51–0.98)	0.012	0.64 (0.45–0.91)	0.018	0.64 (0.45–0.93)
BMI(kg/m^2^)	0.017	0.95 (0.92–0.99)	0.275	0.98 (0.93–1.02)	0.299	0.98 (0.93–1.02)
Systolic blood pressure	0.395	1.00 (0.99–1.01)				
Heart rate	0.275	0.99 (0.98–1.01)				
Dyslipidemia	0.505	0.88 (0.61–1.28)				
Diabetes mellitus	0.317	0.83 (0.58–1.20)				
Hyperuricemia	0.381	1.21 (0.79–1.85)				
Atrial fibrillation	0.101	1.33 (0.95–1.86)				
Coronary artery disease	0.327	1.22 (0.82–1.83)				
COPD	0.192	1.46 (0.83–2.60)				
Malignant tumor	0.593	1.17 (0.66–2.07)				
Albumin	< 0.001	0.42 (0.30–0.59)	< 0.001	0.49 (0.34–0.71)	0.001	0.51 (0.34–0.77)
Hemoglobin (g/dL)	< 0.001	0.87 (0.80–0.94)	0.760	0.98 (0.90–1.08)	0.852	0.99 (0.90–1.09)
eGFR (ml/min/1.73m^2^)	< 0.001	0.98 (0.97–0.99)	0.027	0.98 (0.97–1.00)	0.011	0.99 (0.98–1.00)
BNP (pg/ml)	0.724	1.00 (1.00–1.00)			0.882	1.00 (1.00–1.00)
ACEi/ARB	0.516	0.89(0.63–1.26)				
Beta blocker	0.642	0.92 (0.66–1.29)				
MRA	0.209	0.81 (0.58–1.13)				
Loop diuretics	0.042	1.64 (1.02–2.63)	0.097	1.52 (0.93–2.49)		
SGLT2i	0.120	0.33 (0.08–1.33)				
Statins	0.468	0.87 (0.59–1.27)				

*ACEi* angiotensin-converting enzyme inhibitor, *ARB* angiotensin receptor blocker, *BMI* body mass index, *BNP* brain natriuretic peptide, *CI* confidence interval, *COPD* chronic obstructive pulmonary disease, *eGFR* estimated glomerular filtration rate, *HR* hazard ratio, *MRA* mineralocorticoid receptor antagonist, *SGLT2i* sodium glucose cotransporter 2 inhibitor

### Subgroup analysis

**Fig.3:**
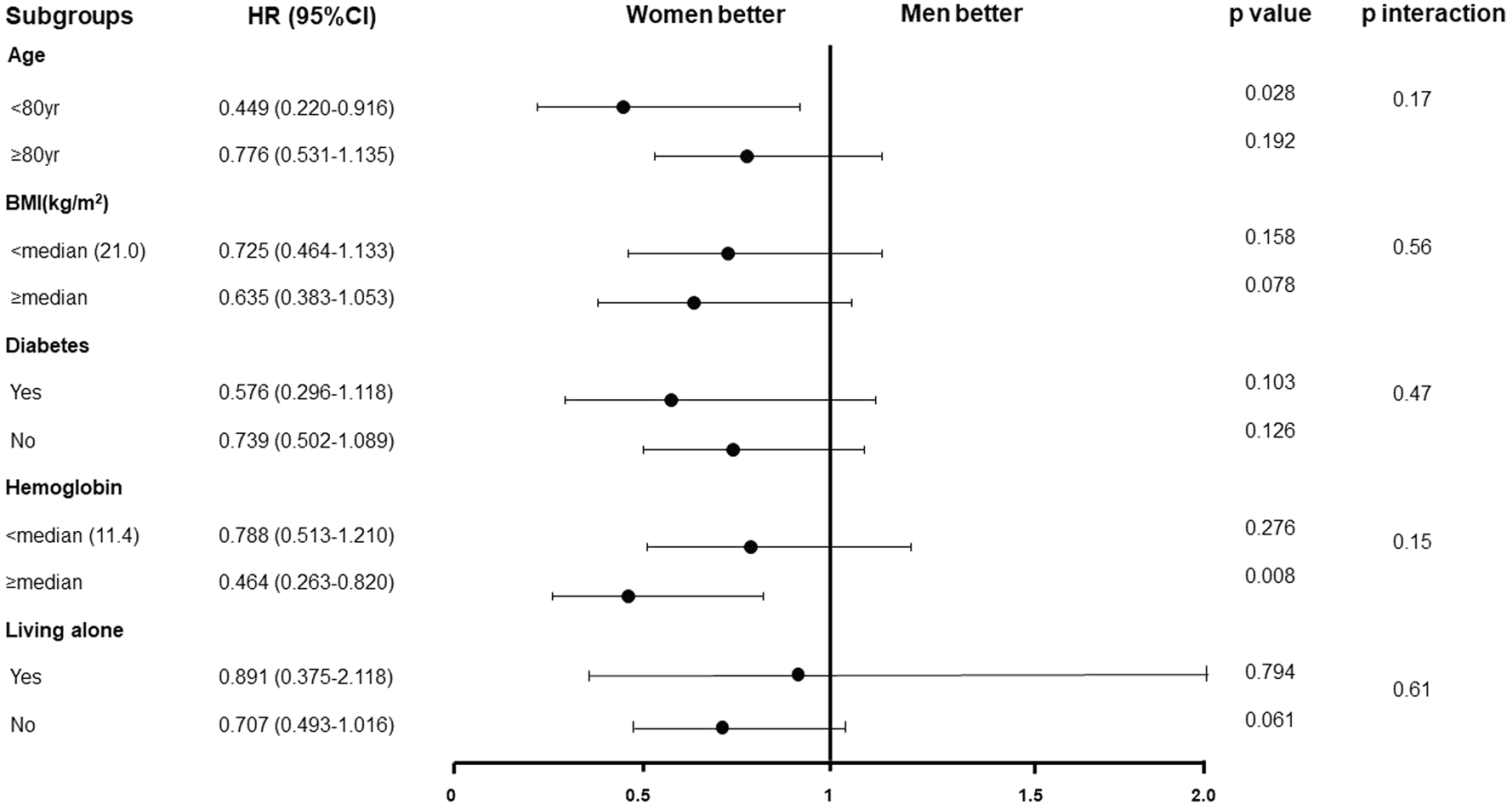
Subgroup analysis on clinically relevant factors. No interactions were observed between the subgroup factors and the risk of women relative to men for the primary outcome

Subgroup analysis of the clinically relevant factors is shown in Fig. 3. No interactions were observed between the subgroup factors and the risk for women relative to men for the primary outcome.

## Discussion

The present study revealed that compared with men, women were older but were less likely to experience an ischemic heart disease, diabetes mellitus, hyperuricemia, and COPD. Furthermore, women were independently associated with a lower risk of cardiovascular death or hospitalization for HF in the Japanese HFpEF cohort.

Previous studies have reported that HFpEF is more frequent in women, and the average age is older in women than in men [8, 9]. In these studies, women had a lower risk of mortality due to HF. However, these results were obtained from studies conducted a few decades ago, and the demographics of the HF population must have been in transition. Recently, a meta-analysis of three major trials (CHARM-Preserved, I-Preserve, and TOPCAT-Americas) revealed that women were older and have a higher frequency of obesity and hypertension; however, the risk of cardiovascular-related death or hospitalization for HF was lower in women compared to men [10].

In Japan, gender differences for long-term outcomes in patients with HFpEF have rarely been reported. The acute decompensated heart failure syndromes (ATTEND) registry was the first major study evaluating the epidemiology of acute HF syndrome in Japan [11]. Gender differences were evaluated regarding anemia and EF in a sub-analysis of the ATTEND registry [12]. In the ATTEND registry, the mean age for women was older than the mean age for men. Comorbidity and medication were not much different from those of our cohort study. Long-term outcome differences between women and men with HFpEF were not directly compared in the ATTEND registry. The Chronic Heart Failure Analysis and Registry in the Tohoku District 2 (CHART–2) study was one of the largest studies in Japan, and gender differences were examined in patients with stage C/D HF [13]. The results showed women were older than men and had a lower prevalence of ischemic heart disease, diabetes, smoking, and cancer; which was consistent with our cohort. On multivariate Cox regression analysis, women had a reduced risk of mortality. However, this result was obtained from the entire HF population, and patients with reduced EF were not excluded. In the Kyoto Congestive Heart Failure registry, women were older than men. Hypertension was more prevalent in women, but ischemic heart disease was less prevalent in women. The risk of all-cause death or hospitalization for HF were significantly lower among women than men after multivariable adjustment [14], but patients with HFrEF were also not excluded. In comparison with the three studies above, patients in our study were older, but comorbidities were not much different. Long-term outcomes were incomparable in the three studies, but our results suggested a higher rate of HFpEF in the future in an aging society. To the best of our knowledge, the present study is the first to describe the risk of cardiovascular-related death or hospitalization for HF in the Japanese HFpEF population.

There are several hypotheses for why women experience better outcomes than men in the HFpEF population. First, women have an ischemic etiology less often than men whereas BNP and EF were not statistically different. In our cohort study, BNP was not an independent predictor for the primary outcome. BNP has been reported to be a prognostic factor for a composite of hospitalization for HF or all-cause mortality in the HFpEF population previously [15]. Recently, the PURSUIT-HFpEF registry showed a NT-pro BNP before discharge ≥ 1611 pg/mL was a useful marker for hospitalization due to CHF, but its hazard ratio after adjusting covariates was 1.00 (95% CI: 1.00–1.00) in patients who were over 80 years old [16]. Patients in our cohort and the PURSUIT-HFpEF registry were older than those of other registries. Elderly patients usually have more non-cardiac comorbidities such as frailty and malignancies, and these comorbidities could have a further impact on the prognosis than BNP or heart failure severities. Since the association between BNP and long-term outcome diminish in older patients with HFpEF, BNP would not be a useful prognostic marker. The lower incidence of ischemic heart disease would be due to estrogen [17] and this is consistent with previous reports [10]. Furthermore, a previous study reported that there was cellular evidence that women possess a possible resistance to cardiomyocytes in the case of myocardial infarction. Another study reported that men have a tenfold higher peri-infarct necrotic index following a fatal myocardial infarction [18]. On the other hand, Aggarwal et al. reported higher mortality and morbidity in women presenting with ACS than men. However, the poor outcome in women could be caused by inappropriate treatments, at least in part [19]. Women were reported to be treated less often by emergent revascularization in myocardial infarction, and were prescribed aspirin, beta-blockers, and statins less often than men. These tendencies were found more often for Black women than others, especially when considering younger patients who were lacking social support, community resources, cognitive deficiencies, and physician follow-up. In Japan, Wada et al. reported that female gender was not an independent predictor for the incidence of major adverse cardiac events after adjusting for age and other variables among patients with ACS [20]. The limited diversity of races or ethnicity in Japan could have attenuated the differences in outcomes between women and men after ACS. Although EF was not statistically different between women and men, the vulnerability for myocardial ischemia in men would result in a higher incidence of cardiovascular death or hospitalization for HF.

Second, there are other unique factors related to women with HFpEF. For example, vascular function contributes to the pathophysiology of HFpEF. A previous study reported that vascular responses to salt intake or angiotensin II were different between women and men [21]. This difference may extend to and have implications for pharmacological therapy in HFpEF. TOPCAT-Americas showed that women treated with spironolactone exhibited a lower risk of cardiovascular-related death, although no effectiveness was observed in men [22]. Moreover, in the PARAGON-HF trial, only women who were treated with ARNI exhibited risk reduction of the primary outcome [5]. In our study, the frequencies of drug use for HF, including angiotensin-converting enzyme inhibitor or angiotensin receptor blocker, β-blocker, mineralocorticoid receptor antagonists, and loop diuretics were not markedly different between women and men. Tolvaptan, that was reported to be useful for preventing HF readmission for HFpEF patients [23], was prescribed more often for men (21.9%) than for women (12.9%). The difference in the efficacy of pharmacological therapy between women and men was suggested in the PARAGON-HF trial, but it remains an unsolved problem and needs further exploration.

Third, we should recognize differences in the social background. A past study reported that living alone was a risk factor for the advanced disability and a poorer prognosis [24]. Recently, living alone after the first HF discharge was reported to be a risk for rehospitalization for HF in men, but not in women [25]. Although HF-ACTION study, which enrolled patients with chronic HF between 2003 and 2007 in a Western country, retrospectively showed that having a partner was not an independent predictor of long-term clinical outcomes [26], the patients in this study were relatively young and self-care disabilities were not conspicuous. In our study, the ratio of living alone was lower for women than in men. Women were supposed to live with their spouses or a family member, and this could be helpful to avoid being socially isolated and avoid worsening HF.

There is no established therapy for the treatment for HFpEF. However, a focus on gender differences may lead to the discovery of a gender-specific HFpEF treatment. It should be not simply a pharmacological approach but should also have certain support systems such as cognitive function support or supportive family members. Longitudinal studies with gender-specific outcomes as the primary aim should be conducted.

### Limitations

There are several limitations in this study. First, this was an observational study. Several confounders that were not measured might affect the patient’s clinical course, and patient backgrounds were not equalized. Second, the number of samples was relatively small; therefore, propensity score matching analysis could not be performed. Third, we did not obtain information about coronary angiography or revascularization. Hao et al. reported that residual stenosis in patients with ischemic HF could aggravate long-term prognosis for patients with LVEF 40–49% (HFmrEF) and LVEF ≥ 50% (HFpEF) patients [27]. Further investigation is needed, including studies on residual coronary stenosis and revascularization.

## Conclusion

Women with HFpEF have a lower risk of cardiovascular death or hospitalization for HF in the Japanese population.
